# A Small Object Detection Method for Oil Leakage Defects in Substations Based on Improved Faster-RCNN

**DOI:** 10.3390/s23177390

**Published:** 2023-08-24

**Authors:** Qiang Yang, Song Ma, Dequan Guo, Ping Wang, Meichen Lin, Yangheng Hu

**Affiliations:** 1 School of Automation, Chengdu University of Information Technology, Chengdu 610225, China; yangqiang@cuit.edu.cn (Q.Y.); masong0007@163.com (S.M.); linlynn98@163.com (M.L.); huyhctu@163.com (Y.H.); 2 Key Laboratory of Natural Disaster Monitoring & Early Warning and Assessment of Jiangxi Province, Jiangxi Normal University, Nanchang 330022, China; 3 School of Network & Communication Engineering, Chengdu Technological University, Chengdu 610031, China; wangpingqqss@126.com

**Keywords:** oil leakage detection, small object detection, substation equipments, faster-RCNN, intelligent inspection robot

## Abstract

Since substations are key parts of power transmission, ensuring the safety of substations involves monitoring whether the substation equipment is in a normal state. Oil leakage detection is one of the necessary daily tasks of substation inspection robots, which can immediately find out whether there is oil leakage in the equipment in operation so as to ensure the service life of the equipment and maintain the safe and stable operation of the system. At present, there are still some challenges in oil leakage detection in substation equipment: there is a lack of a more accurate method of detecting oil leakage in small objects, and there is no combination of intelligent inspection robots to assist substation inspection workers in judging oil leakage accidents. To address these issues, this paper proposes a small object detection method for oil leakage defects in substations. This paper proposes a small object detection method for oil leakage defects in substations, which is based on the feature extraction network Resnet-101 of the Faster-RCNN model for improvement. In order to decrease the loss of information in the original image, especially for small objects, this method is developed by canceling the downsampling operation and replacing the large convolutional kernel with a small convolutional kernel. In addition, the method proposed in this paper is combined with an intelligent inspection robot, and an oil leakage decision-making scheme is designed, which can provide substation equipment oil leakage maintenance recommendations for substation workers to deal with oil leakage accidents. Finally, the experimental validation of real substation oil leakage image collection is carried out by the intelligent inspection robot equipped with a camera. The experimental results show that the proposed FRRNet101-c model in this paper has the best performance for oil leakage detection in substation equipment compared with several baseline models, improving the Mean Average Precision (mAP) by 6.3%, especially in detecting small objects, which has improved by 12%.

## 1. Introduction

Substations are important for the stable operation of power systems. In the power system, the main task of the substation is to control the generation, transmission and distribution of electricity [1]. During operation, any fault will directly affect the overall operation of the power system and pose a huge threat to its safety. Thus, it is necessary to ensure the safe and stable operation of the substation [2]. At present, the safety inspection of substations is attracting the attention of a number of academics. The traditional manual inspection method relies on manual monitoring of substations around the clock. The manual inspection needs to question the physical condition of the substation and the subjective judgement of the workers, whose overload and inexperience will prevent them from effectively identifying harmful conditions in the substation [3]. As a result, smart substations are now increasingly using inspection robots to replace manual inspections. The application of substation inspection robots is expanding as substation inspection intelligence continues to develop [4,5,6]. Due to the fact that the inspection robot is equipped with numerous sensors to effectively monitor the state of the substation equipment, it can replace the traditional manual inspection method [7]. However, although the majority of routine inspection tasks can be completed, some potential equipment defects in the substation have not been found in time. For example, the automatic detection of oil leakage defects in substation equipment by inspection robots can lead to serious safety incidents such as fires, which can seriously threaten the safety of the substation. Therefore, there is still great challenge in designing an efficient and reliable method for oil leakage detection in substation equipment in combination with an inspection robot.

In recent years, the research on object detection methods for substation inspection robots has mainly focused on substation high-voltage circuit breakers and various types of instrument detection, and the object detection methods for substation oil leakage defects are still in the initial stage [8]. The object detection methods can be divided into two categories. One category is the traditional object detection methods, which mainly use sliding windows for region selection, Scale-Invariant Feature Transform (SIFT) [9]/ Histogram of Oriented Gradient (HOG) [10] for feature extraction, and Support Vector Machine (SVM) [11]/Adaboost [12]/Bayesian [13] as classifiers for object detection in images. However, traditional object detection has the following disadvantages: (i) the sliding window selection strategy uses untargeted, high-time-complexity, and redundant windows and (ii) the manually designed features have poor robustness. With the development of artificial intelligence technology, another category of deep learning methods has achieved remarkable performance in object detection [14,15]. The deep-learning-based object detection method improves on the main problems of the sliding window strategy in traditional object detection methods as well as the poor robustness of manually designed features. Due to the excellent performance of deep-learning-based object detection methods, they are popular in a wide range of industries and are widely used in areas such as intelligent transportation [16], aerospace [17], health care [18], and industrial detection [19]. However, the use of deep learning methods on substation inspection robots and the detection of various types of power equipment defects based on deep learning are relatively little researched. Therefore, in order to apply deep-learning-based object defect detection methods to inspection robots, it is necessary to research the detection of oil leakage defects in substation equipment.

In order to solve the problem of oil leakage detection in substation equipment with small objects and combine it with intelligent inspection robots to provide inspection workers with a decision-making method to deal with oil leakage accidents, this paper proposes a small object detection method for oil leakage defects in substations. In this paper, the Faster-RCNN [20] network is selected and improved for the detection of oil leaks in substation equipment by inspection robots. An oil leakage defect detection model is finally designed for substation equipment using inspection robots, which is called the FRRNet101-c model. The FRRNet101-c model integrates the advantages of the ResNet [21] and VGGNet [22] models. The advantage of the ResNet model is mainly its the residual structure, which avoids the problem of vanishing gradients when the layers are deeper. The advantage of the VGGNet model is the small convolutional kernel instead of a large convolutional kernel to improve the performance of the model. Hence, through the elimination of the downsampling operation and the replacement of the convolutional kernel, it can effectively reduce the loss of information in the original image, especially for small objects. In addition, the proposed method can be used to give advice on the maintenance of substation equipment after the detection of an oil leakage in the substation. The experimental results show that the proposed method in this paper has high detection accuracy and efficiency, especially for small objects, compared to several baseline models. Meanwhile, it also shows remarkable decision-making performance in the actual oil leakage detection and maintenance of substation equipment.

The contributions of this paper are summarized as follows:

(1) A small object detection method is proposed for oil leakage defects in substations based on improved Faster-RCNN. Canceling the downsampling operation and replacing the large convolutional kernel with a small one can reduce the loss of information in the original image, especially for small objects.
(2) The proposed method designs an oil leakage decision-making scheme to provide recommendations for oil leakage maintenance in substation equipment, which provides guiding information for substation workers to deal with oil leakage accidents.

The rest of this paper is organized as follows. Section 2 introduces the work related to this paper. Section 3 describes the method proposed in this paper in detail. Section 4 provides an experimental validation of the method proposed in this paper. Finally, the conclusions and future work are presented in Section 5.

## 2. Related Works

In substations, there are many oil-filled equipment, and these oil-filled equipment are affected by various complex factors such as their own manufacturing quality, possible problems during transportation and installation, long-term operation, etc., which greatly affect the safe operation of the substation [23]. Therefore, it is extremely important to detect equipment oil leakage defects and give corresponding alarms in a timely manner. The current methods for detecting oil leakage in substation equipment include manual inspection methods, image processing methods, and deep learning methods.

The method of manual inspection is still the main strategy of oil leakage detection for oil-filled equipment in substations, in which the operation and maintenance personnel perform a visual inspection of each device one by one for high-voltage-level equipment, often with the help of binoculars, to detect oil leakages in power equipment [24,25]. Ref. [24] used remote sensing technology to analyze the soil, water, and other image features around the equipment to manually determine where the equipment was leaking oil. Ref. [25] used synthetic aperture radar (SAR) technology to analyze water, vegetation, and other features around the equipment to determine where the equipment was leaking oil. There is still some room for improvement in these methods. For example, workers need to check the oil-filled equipment one by one, which is time-consuming and labor-intensive. The results of the inspection and judgment of different operation and maintenance personnel will vary, making it difficult to accurately determine the oil leakage points and the extent of oil leakage. With the widespread use of image processing technology in power systems, an oil leakage detection method for power equipment through image processing has been studied [26]. This category mainly uses complex processing of images to detect oil leakage from substation equipment. Ref. [27] designed a method for detecting oil leakage from power transformers under solar irradiation. In Ref. [28], image processing was used to detect transformer oil leakage, and used the relationship between saturation and intensity in the hue–saturation–intensity color space. Ref. [29] detected oil leakage from transformers by distinguishing between sample images and monitoring images to obtain anomalous areas, followed by observing the color change in the anomalous areas utilizing H-S color histograms. Although these methods can solve the detection of oil leakage in some of the current equipment in substations, they also have certain shortcomings, such as a low level of intelligence in automatic feature extraction.

With the development of artificial intelligence technology, the emergence of deep learning methods has attracted the attention of many scholars in order to further enhance the intelligence of oil leakage detection in substation equipment [30]. Deep learning methods are a primarily data-driven form of automatic feature extraction, which does not rely on manual feature extraction, and the extracted abstract features are more robust, generalize better, and can be trained end-to-end, among other benefits. Some deep-learning-based oil leakage detection methods has been researched for substation equipment. In Ref. [31], a deep convolutional neural network (DCNN) method based on transfer learning was developed to detect insulating oil leakage from substation equipment. It provides better performance than traditional image processing methods, and the proposed method is also used in substation image monitoring systems. Ref. [32] proposed a fusion of a dual-attention mechanism and a residual network to improve the U-Net model for transformer oil leakage detection. The proposed method can identify the oil leakage area with high accuracy. Ref. [33] proposed the generation of synthetic oil leakage images based on generative adversarial networks (CycleGAN), in which the original image was combined with the synthetic image and the YOLOv4 network was trained, and this method can be used for substation equipment oil leakage detection. The above-mentioned deep-learning-based approaches for oil leakage detection in substation equipment have achieved better performance in terms of accuracy. However, these deep-learning-based approaches to substation oil leakage detection have not been combined with intelligent inspection robots in real-world scenarios to further improve intelligence. To this end, this paper proposes a highly efficient and more reliable oil leakage detection method for substation equipment and an oil leakage decision-making scheme combined with an intelligent inspection robot.

## 3. The Proposed Oil Leakage Detection Method

**Figure 1:**
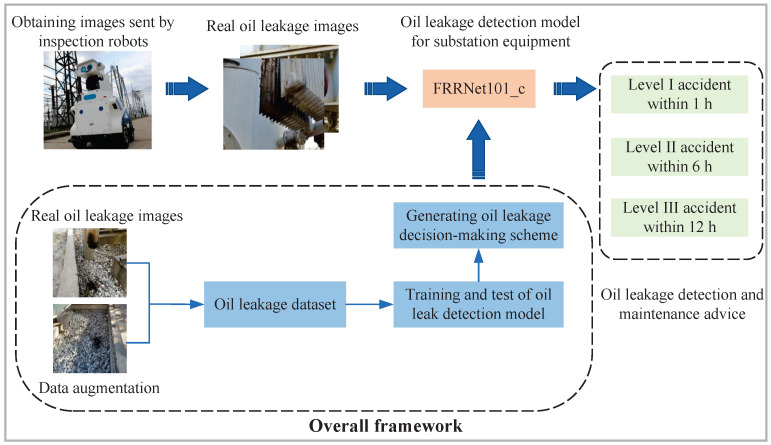
The overall framework for oil leakage detection in substation equipment.

In this paper, deep-learning-based object detection techniques are applied to the detection of oil leakage in substation equipment. Firstly, images of oil leakages in substation equipment are collected by inspection robots. Then, the collected images of oil leakage in substation equipment are detected using the best-performing oil leakage detection model after training and testing. Finally, the FRRNet101-c model proposed in this paper detects oil leakage from substation equipment and gives maintenance suggestions. In this case, in the FRRNet101-c model, the dataset consists of real substation oil leakage images and data-augmented images. After that, the FRRNet101-c model for substation equipment oil leakage detection is trained and tested, and a decision solution for oil leakage is generated. The overall framework for oil leakage detection in substation equipment is shown in Figure 1.

### 3.1. Image Acquisition Device

**Figure 2:**
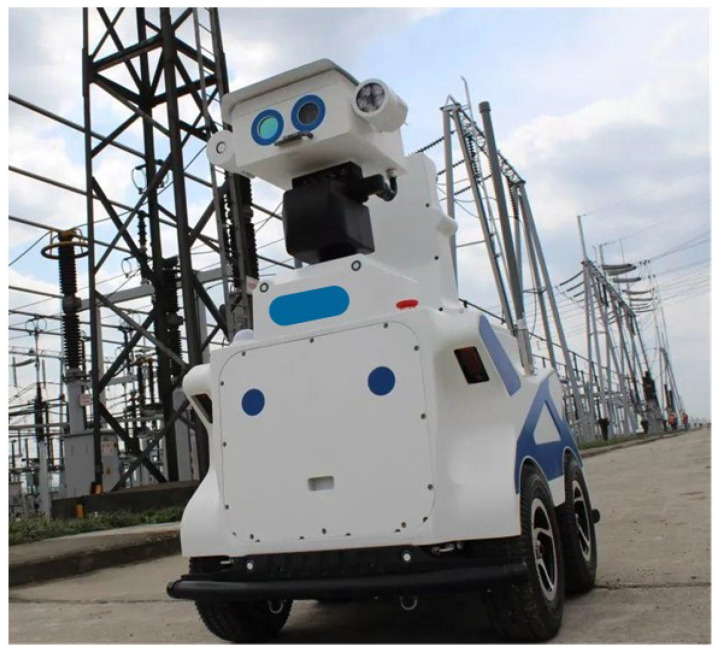
Substation inspection robot from a Chinese company.

**Figure 3:**
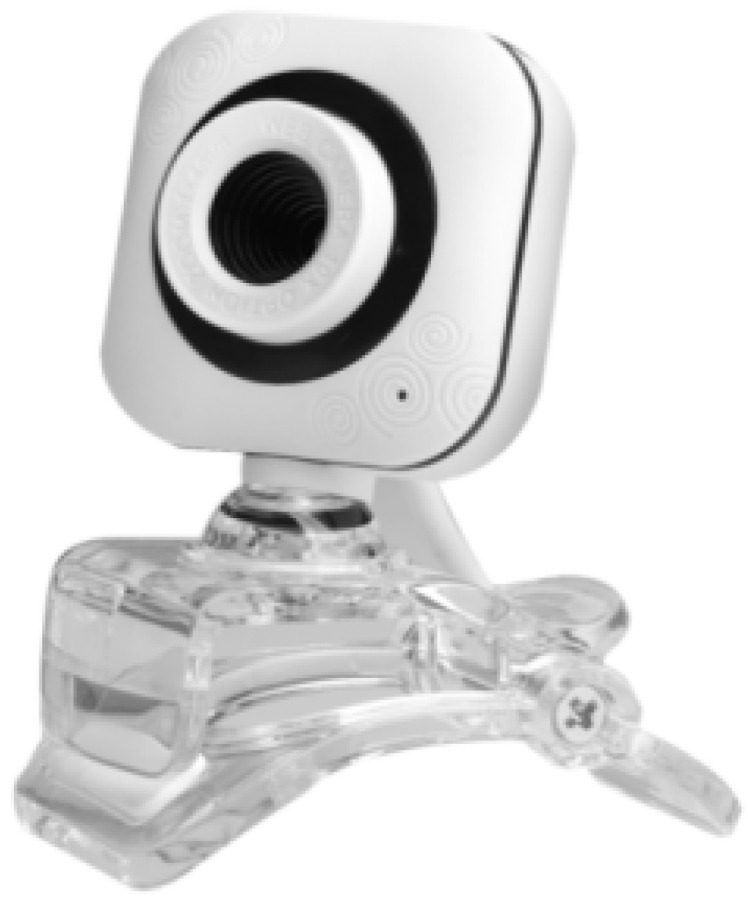
USB 2.0 camera.

To collect images of oil leakages in substation equipment, this paper uses an intelligent inspection robot from a Chinese robot company with a USB 2.0 camera as the image acquisition module. In the market, various substation inspection robots are different. The intelligent inspection robot mainly consists of core equipment such as a camera, intelligent head, lidar, robot core control chassis, emergency brake module, and four-wheel drive chassis. The intelligent inspection robot is shown in Figure 2. The USB 2.0 camera is shown in Figure 3.

The substation inspection robot performs automatic setting of inspection tasks, automatic recording of inspection results, and information interaction with the existing information system of the substation. The substation inspection robot not only replaces traditional manual inspection but also has the ability to diagnose faults in the operation of substation equipment. In addition, the substation inspection robot effectively improves the quality and efficiency of inspection, reduces the labor intensity of manual inspection and the safety risks associated with inspection in harsh climatic conditions, and further ensures the safety of substation workers and substations.

### 3.2. Oil Leakage Detection Model

#### 3.2.1. Selection of Oil Leakage Detection Model

Due to the fact that there is no deep learning network dedicated to the detection of oil leakages in substation equipment, this section will therefore focus on the current deep-learning-based object detection networks and combine them with the characteristics of the oil leakage defects in the substation equipment itself, so as to find a suitable network for the detection of oil leakage defects in substation equipment. Although various networks continue to be introduced, the mainstream object detection networks are still based on convolutional neural networks (CNN) and R-CNN classified object detection networks (R-CNN [34], SPP-NET [35], Fast R-CNN [36], Faster R-CNN [37]), as well as object detection models that transform object detection into a regression problem, represented by SSD. In this paper, a systematic analysis of the current Faster R-CNN, R-FCN [38], and SSD [39] object detection networks is presented from the following three points.

(1) The Mean Average Precision (mAP) of the three object detection networks is compared on the PASCAL VOC dataset and the COCO dataset, as well as their detection efficiency.
(2) The detection accuracy is compared for different-sized targets based on different feature extraction networks.
(3) Based on the different feature extraction networks, the overall model detection accuracy and efficiency and the size of their models are compared.

The comparison of these models is carried out on the above three points. The results of the comparative analysis of the three models are shown in the experimental analysis section. Finally, Faster-RCNN-Resnet-101 is selected in this paper as the base model for oil leakage detection in substation equipment using inspection robots, and further research on network accuracy and efficiency improvement is carried out by combining the characteristics of oil leakage defects in actual substation equipment.

#### 3.2.2. FRRNet101-c Model Architecture

Oil leakage detection in substation equipment is based on the Faster-RCNN-ResNet101 model and improvements to it. Since in CNNs the shallow network usually contains more image information, if the original input image is downsampled, some information in the original image will be lost, especially information about small objects. Therefore, this can be avoided by removing some of the downsampling operations from the network while replacing large convolutional kernels with multiple small convolutional kernels, which can effectively improve the use of the original image information, especially for small objects.

In this paper, the feature extraction network ResNet-101 in the basic model is improved. Based on ResNet-101, the first two layers of downsampling operations are sequentially removed. With the removal of the two downsampling operations, there is an increase in the amount of data and computation in the network. To address this shortcoming, it is possible to replace the large convolution kernel by stacking multiple small convolution kernels, where the first 7 × 7 convolution kernel is replaced with three 3 × 3 convolutions. This ensures that the perceptual field remains the same before and after the improvement, while at the same time increasing the depth of the network, improving its effectiveness to a certain extent and reducing the number of computational parameters. Thus, the model proposed in this paper is called the FRRNet101-c model. The FRRNet101-c (layer 103) model’s layer structure is shown in detail in Table 1.

The formula for the convolution layer is shown in Equation (1).

(1) $$conv_{output}=f\left(W_{X}^{T}+b\right)$$

where f() is the activation function, *X* is input data, and *W* and *b* are the weights and bias parameters, respectively. The activation function is selected as RELU [40]. The formula is shown in Equation (2).

(2) f(x)=max(0,x)

The derivation formula is shown in Equation (3).

(3) $$f^{{}^{\prime }}\left(x\right)=\left\{\begin{array}{cc} 1 & x>0\\ 0 & x\leq 0 \end{array}\right.$$

The formula of the downsampling layer is expressed by average pooling as shown in Equation (4).

(4) $$averagepooling_{output}=\frac{1}{\left|R_{ij}\right|}\sum_{\left(i,j\right)\in R_{ij}}x_{kpq}$$

where $x_{kpq}$ is an element at (p,q) in the area of pooling and $\left|R_{ij}\right|$ denotes the size of the pooling region.

The input to the full connection layer is a vector. The vector obtained via rasterization is connected to the full connection layer, and finally the classification result of the classifier is obtained via the Softmax classifier. The Softmax classifier accepts 4096 dimensions of input data, and the output result is *n* dimensions (*n* is the number of equipment leakage defect types), which represents the confidence degree of the input sample corresponding to n equipment oil leakage defect categories and then takes the category with its maximum value as the classification result.

The formula for the Softmax classifier is shown in Equation (5).

(5) $$f{\left(z\right)}_{j}=\frac{e^{z_{i}}}{\sum_{k=1}^{K}e^{z_{k}}}$$

where j=1,2,⋯,K, *K* is the number of classes, the value of which is *n* (the number of 232 equipment leakage defects types), $z=W_{x+b}^{T}$, *W*, and *b* are the parameters of Softmax, and *x* is the input feature and has 4096 dimensions.

In summary, the FRRNet101-c (layer 103) model is constructed as follows:

1. The basic model is determined. In this paper, through the analysis of the experiments, the selected basic model is the Faster-RCNN-ResNet101 (FRRNet101) model.
2. In the basic FRRNet101 model, the step size of the first convolutional layer is replaced, which cancels the first downsampling operation.
3. Following step 2, the max-pooling layer in the basic FRRNet101 model is cancelled, which is the cancellation of the second downsampling operation.
4. After canceling the two downsampling operations, the three 3 × 3 convolutional kernels replace the first 7 × 7 convolutional kernel in the middle of the base FRRNet101 model to obtain the FRRNet101-c model in this paper.

### 3.3. Oil Leakage Decision-Making Scheme

The use of object detection technology in deep learning to detect information about oil leakage from substation equipment and to achieve the intelligent detection of oil leakage from substation equipment can replace manual detection. However, this method still requires substation inspection workers to look for oil leakage points in a specific way at a specific time, and dealing with oil leakage accidents is fuzzy. If automated tools can be used instead of workers to understand the current inspection work environment and obtain oil leakage detection results, this will help to improve the intelligence of oil leakage detection in substation equipment. Therefore, in order to further improve the intelligence of substation equipment oil leakage detection, this paper proposes the design of an oil leakage decision scheme to generate substation equipment oil leakage maintenance opinions, so as to reduce the workers’ substation inspection task.

The oil leakage decision-making scheme is mainly based on the equipment oil leakage information obtained from object detection and the relevant regulations of the substation, generating maintenance opinions about substation equipment oil leakage.

**Figure 4:**
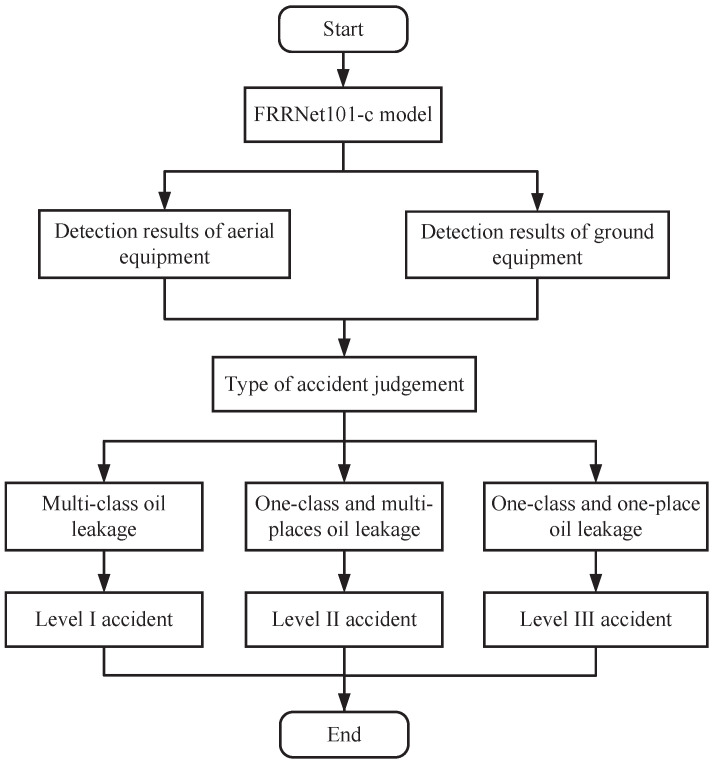
The substation equipment oil leakage decision-making scheme flow chart.

This paper focuses on the detection and study of six common equipment oil leakage situations in substation scenarios, including tanker oil seepage, heat sink oil seepage, valve oil seepage, stone on oil, column device oil leakage, and ground on oil. The automatic generation of substation equipment maintenance recommendations is based on the FRRNet101-c model’s results. The six common types of substation oil leakage defects processed via the model are divided into three types of maintenance suggestions: “Level I accident”, “Level II accident”, and “Level III accident”. The flowchart for the substation equipment oil leakage decision-making scheme is shown in Figure 4.

In this case, for multi-class equipment oil leakage, such as “column oil leakage”, “valve oil leakage”, “column oil leakage”, and so on, which appear at the same time, substation equipment maintenance is recommended for a “level I accident”, which means great attention should be paid to multi-class equipment oil leakage and emergency maintenance should be performed immediately within 1 h. In addition, when there is a class of equipment with multiple oil leakage accidents, such as “multiple oil leakage from valves”, the substation equipment maintenance suggestion is a “Level II accident”, which means that the cause of the oil leakage needs to be discovered and the equipment should be maintained within 6 h. Finally, if there is only one oil leakage incident in a class of equipment, for example, “one oil leakage in a valve”, then the substation equipment maintenance suggestion is a “Level III accident”, which means that the cause of the leakage needs to be discovered and the equipment should be maintained within 12 h.

## 4. Experimental Results and Analysis

In this section, the proposed oil leakage detection method for substation equipment is analyzed experimentally, mainly for the selection of the base oil leakage detection model, the improvement of the base model, and the implementation of the oil leakage decision-making scheme, respectively.

### 4.1. Experiments Environment and Dataset

**Figure 5:**
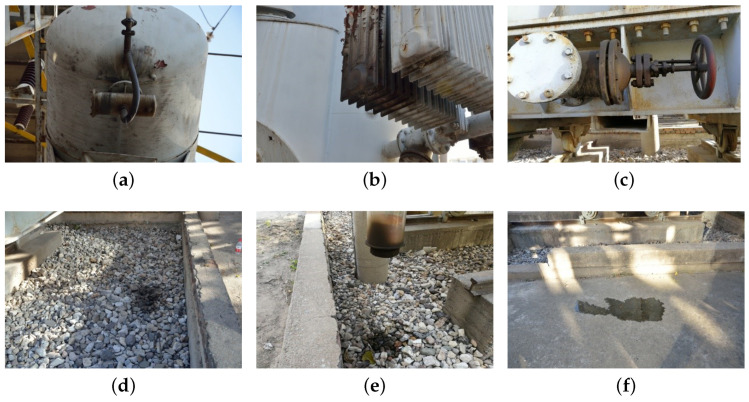
The six common types of equipment oil leakage in substations: (**a**) oil leakage from tanks; (**b**) oil leakage from heat sinks; (**c**) oil leakage from valves; (**d**) oil on stones; (**e**) oil leakage from columns; (**f**) oil on ground.

The experimental data in this paper were derived from a public dataset and a substation equipment oil leakage defects dataset collected in a real scenario. The public datasets used in this paper are the PASCAL VOC dataset and the COCO dataset, which were used to validate the chosen base models, Faster R-CNN, R-FCN, and SSD, for comparison. The real-scenario substation equipment oil leakage detection dataset consists of six common equipment oil leakages in the substation, which include oil leakage from tanks, oil leakage from heat sinks, oil leakage from valves, oil leakage from stones, oil leakage from column devices, and oil leakage from the ground, as shown in Figure 5. The proportion of labels for the six categories of equipment is 1:0.4:1.3:0.6:0.8:0.9. The data collection method for the real-scenario substation equipment oil leakage dataset used substation inspection robots with cameras to collect images of oil leakage defects with different lighting, scenes, and angles in each substation. The original image size was collected as a different pixel value image with a maximum length of 2560 and a maximum width of 1920. Due to the small amount of oil leakage data from equipment in real substations, a total of 438 images were collected. Therefore, a total of 438 real substation equipment oil leakage images are used as the original dataset, of which 399 are the training set and 39 are the test set, and the ratio of the training set to the test set is close to 9:1.

For implementing the methodology of this paper, all experiments were performed on the Ubuntu MATE operating system and the TensorFlow [41] deep learning framework. The computer system’s processor and RAM are 3.10 GHz i5-12500H (CPU) and 32.00 GB (RAM), respectively. In order to implement the functional module of oil leakage defect detection for the substation inspection robot, the hardware platform uses the Raspberry Pi 3B+ development board.

### 4.2. Data Pre-Processing and Augmentation

Because there are no public datasets related to oil leakage detection in substation equipment, this paper requires data pre-processing of the images collected via the intelligent inspection robot as a way to build the dataset for the study of this paper’s methodology. To produce a standard object detection dataset, the images were normalized to a pixel size of 640 × 480 and labeled manually by hand. The six types of images collected were collated and labeled. The image labeling tool used in this paper was LabelImg.

After labeling the dataset, it was found that the number of target boxes in some categories was too small, resulting in an uneven sample, so targeted data augmentation was adopted. The analysis of images with fewer categories of oil seepage from heat sinks and oil on stones revealed that these datasets were more influenced by the angle of the shot and the lighting and had more small objects. Therefore, data augmentation through changing the brightness of the image and scaling the image size was used to increase the number of objects and enrich their information at the same time. Although the first data augmentation process was performed on the samples, 560 images were still not enough for the training of the model through deep learning, which could easily lead to overfitting of the trained model and insufficient generalization ability, thus seriously affecting the detection accuracy of the model. Therefore, the second data augmentation process for each category by means of rotation, traverse, scaling, flipping, noise addition, and light and dark changes was adopted. The proportion of labels for the six categories of equipment was 1:0.9:1.1:0.9:1:0.9.

Finally, for the second data augmentation process, keeping the ratio of each type of label constant, the total number of images reached 3300, with 3000 in the training set and 300 in the test set. The second data augmentation of the dataset was used for model training and testing of the data. The comparison of the substation equipment oil leakage dataset before and after data augmentation is shown in Table 2.

### 4.3. Analysis of the Selection of Base Oil Leak Detection Models

**Figure 6:**
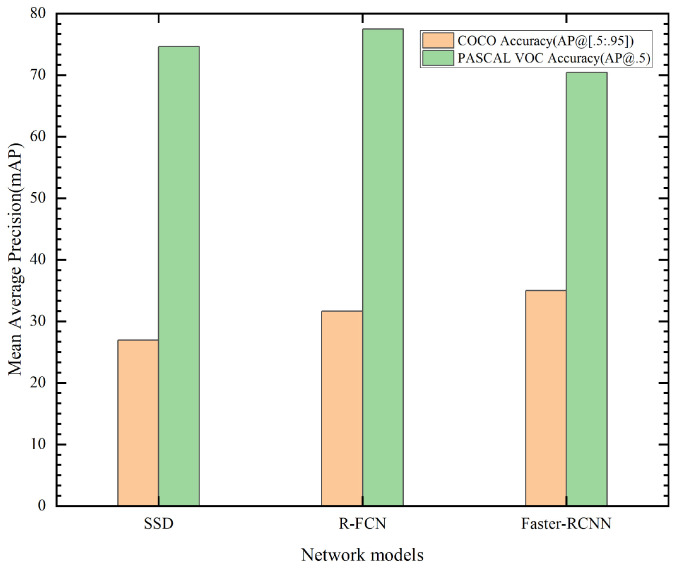
Three models on the public dataset for mAP comparison.

**Figure 7:**
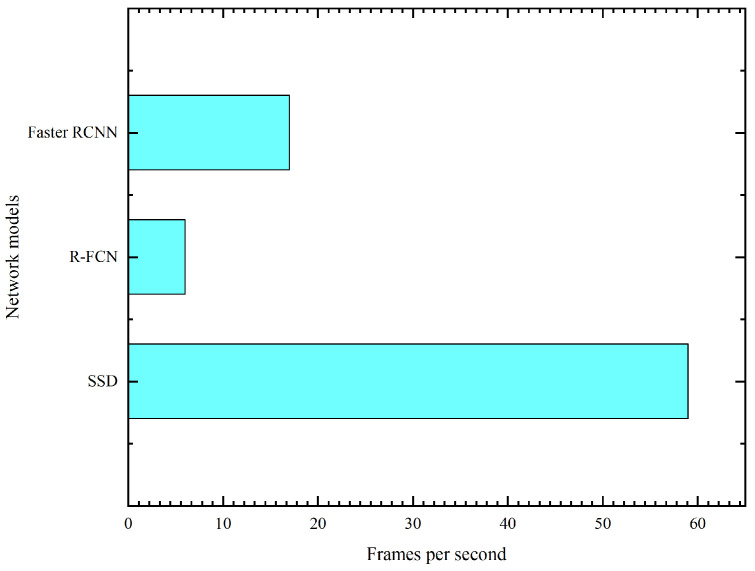
The frame rate comparison of the three models.

This section provides a comparative analysis and summary of the Faster R-CNN, R-FCN, and SSD. Firstly, the mAP and detection efficiency of the models tested on the PASCAL VOC dataset as well as on the COCO dataset were compared. The results of three models using a public dataset for mAP comparison are shown in Figure 6. The frame rate comparison of the three models is shown in Figure 7. From Figure 6, it can be seen that Faster RCNN, RFCN, and SSD all show better accuracy on the PASCAL VOC dataset, while R-FCN has the best accuracy. In the COCO dataset, there was a large disparity between the test results of the three models relative to the PASCAL VOC dataset, where Faster-RCNN (using ResNet as a feature extractor) had the highest accuracy (mAP @ [.5:.95]). In Figure 7, the Faster-RCNN and R-FCN models show better performance in terms of frame rate.

**Figure 8:**
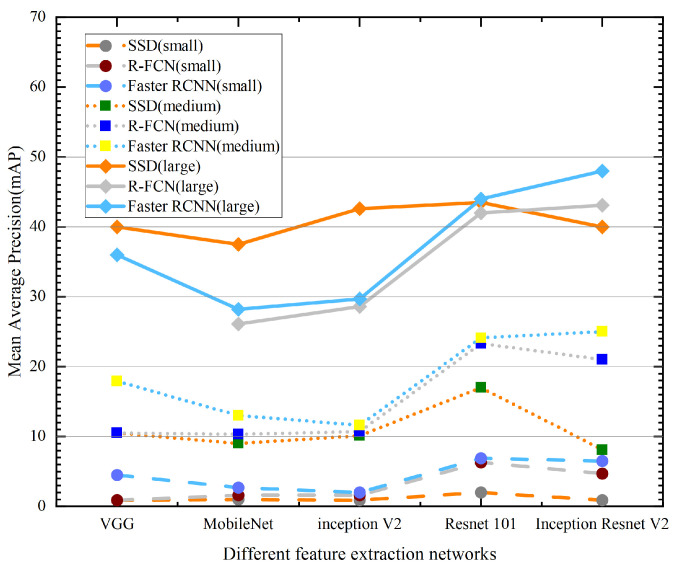
The mAP of different feature extraction networks for object detection with different object sizes.

The feature extraction network is mainly used to extract the feature map from the input image. Different feature extraction networks are different in the amount of feature map information extracted from the input image, and their own accuracy leads to different mAPs in the model. In this paper, based on VGG, MobileNet, Inception-V2, Resnet-101, and Inception-Resnet-V2, feature extraction networks were tested and compared on the MS COCO dataset for different object sizes. In the MS COCO dataset, objects with an area smaller than 32 × 32 were defined as small objects, objects with an area larger than 32 × 32 and smaller than 96 × 96 were defined as medium objects, and objects with an area larger than 96 × 96 were defined as large objects. The mAPs of different feature extraction networks for object detection with different object sizes are shown in Figure 8. From Figure 8, both the Resnet-101 and Inception-Resnet-V2 feature extraction networks showed the best results for all three object test mAPs with different object sizes. In particular, for the Faster-RCNN model, the Resnet-101 feature extraction network achieved 44.0%, 24.1%, and 6.9% mAP for large, medium, and small objects, respectively. The Inception-Resnet-V2 feature extraction network achieved 48%, 25%, and 6.5% mAP for large, medium, and small objects, respectively.

From the above experimental analyses, the Faster-RCNN-Resnet-101 and Faster-RCNN-Inception-Resnet-V2 models were used as the base models for further comparison of oil leakage detection in substation equipment using inspection robots. The comparison results of Faster-RCNN-Resnet-101 and Faster-RCNN-Inception-Resnet-V2 in terms of model size, speed, and mAP are shown in Table 3. In Table 3, the Faster-RCNN-Resnet101 has a lower size of 187 MB relative to the Faster-RCNN-Inception-Resnet-V2 model, which is more conducive to hardware platform portability, and the mAP obtained on the COCO dataset for the two models varies by only 5%. In addition, Faster-RCNN-Resnet-101 has a close to six-fold improvement in detection speed compared to the Faster-RCNN-Inception-Resnet V2 model, which is more in line with the real-time requirements of embedded systems.

In summary, the mAPs of the Faster-RCNN-Resnet-101 and Faster-RCNN- Inception-Resnet-V2 models differ only by 5%, and the Faster-RCNN-Resnet-101 has greater advantages in terms of model size and speed. Therefore, the Faster-RCNN-Resnet-101 was selected as the basic model for oil leakage detection in substation equipment using inspection robots, and further model improvement and enhancement studies were performed.

### 4.4. The Proposed FRRNet101-c Model Validation

#### 4.4.1. Model Training and Evaluation Indicators

In the training procedure, both the FRRNet101-c model proposed in this paper and the comparison model were set with the same dataset, number of training iterations, and training parameters. In this case, the learning rate was set to 0.0003, the number of training iterations was 200,000, and the batch images were 2, all of which were computed using the GPU to accelerate the model training and testing. To evaluate the effectiveness and performance of the model detection metrics, the most common object detection metrics AP and mAP were used, of which AP is Average Precision and mAP is Mean Average Precision. The mAP formula is shown in Equation (6).

(6) $$mAP=\int_{0}^{1}P\left(R\right)dR$$

where *P* is accuracy and *R* is recall.

#### 4.4.2. Model Validation

**Figure 9:**
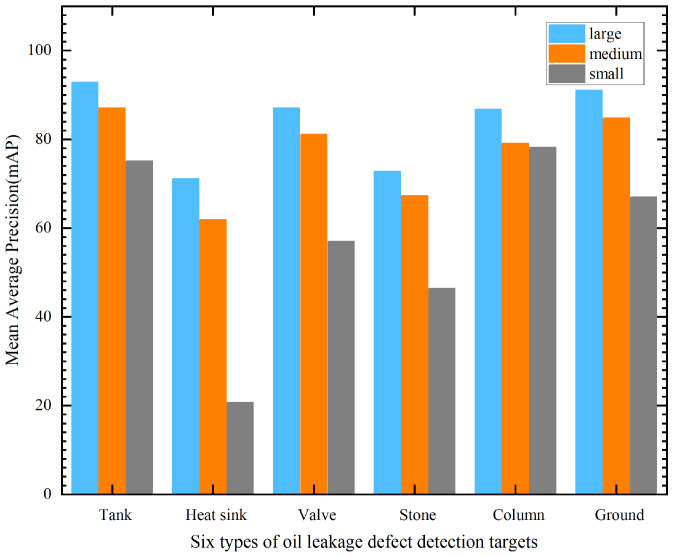
The mAP of oil leakage defects in substation equipment with different object sizes.

Based on the analysis and screening in the previous section, the FRRNet101 (Faster-RCNN-Resnet-101) model has outstanding performance in terms of detection accuracy and detection efficiency and has the advantages of a relatively moderate model size. Therefore, in this section, a reliable and efficient oil leakage detection model for substation equipment is proposed based on the improvement in the FRRNet101 model structure, which is named the FRRNet101-c model. Firstly, the performance of the FRRNet101 model was tested using real collected images of oil leakages from six types of substation equipment. The mAP for oil leakage defects in substation equipment with different object sizes is shown in Figure 9. From the test results of mAP for substation equipment oil leakage defects with different object sizes in Figure 9, it is shown that the FRRNet101 model obtains a better mAP for large and medium objects and achieves the highest mAPs of 92.8% and 87.1% for large and medium objects, respectively. However, for the detection of oil leakage defects in substation equipment with six smaller objects, the lowest mAP was 20.7%. Therefore, the mAP of the FRRNet101 model for small object detection needs to be improved.

In order to further improve the model’s mAP and detection effectiveness, the following improvements were made to the feature extraction network ResNet-101 in the FRRNet101 model:

(1) FRRNet101-aug: To validate the effect of data enhancement, the FRRNet101 model was trained for validation using the original data. The FRRNet101-aug model was trained for validation using the data after the second data augmentation, including the following improved FRRNet101-a, FRRNet101-b, and FRRNet101-c models.
(2) FRRNet101-a: Based on the FRRNet101 model structure, the step size of the first convolutional layer stride = 2 was replaced with stride = 1, which means that the first downsampling operation was cancelled. The reason for this is to reduce the loss of information from the original image, especially for small objects.
(3) FRRNet101-b: The max-pooling layer of the feature extraction network ResNet-101 was removed based on the structure of FRRNet101-a, which means that the second downsampling operation was cancelled. In order to obtain richer image information and resolution, the performance of the model was improved.
(4) FRRNet101-c (ours): On the basis of the structure of FRRNet101-b, the first 7 × 7 convolutional kernel was replaced with three 3 × 3 convolutional kernels. This structure ensures that the perceptual field remains the same before and after the improvement and also improves the depth of the model, which improves the model to a certain extent and reduces the number of computational parameters.

The model was trained and tested, and the experimental comparison of training time consumed is shown in Table 4. The experiments show that the FRRNet101-aug model with data augmentation for oil leakage detection in substation equipment achieved a detection mAP of 91.54% during training and 89.13% during testing, but the training time increased. Compared with the FRRNet101-aug, FRRNet101-a, and FRRNet101-b models, the proposed FRRNet101-c model in this paper improved the mAP detected during training by 6.22%, 4.69%, and 1%, respectively, and the mAP detected during testing by 6.3%, 4.17%, and 0.91%, respectively. However, the proposed FRRNet101-a model has paid a price in terms of training time consumed. Generally speaking, the proposed FRRNet101-a model in this paper cancelled the downsampling operation and the large convolutional kernel was replaced with multiple small convolutional kernels, which effectively improved the model’s effectiveness in detecting the accuracy of oil leakage in substation equipment.

**Figure 10:**
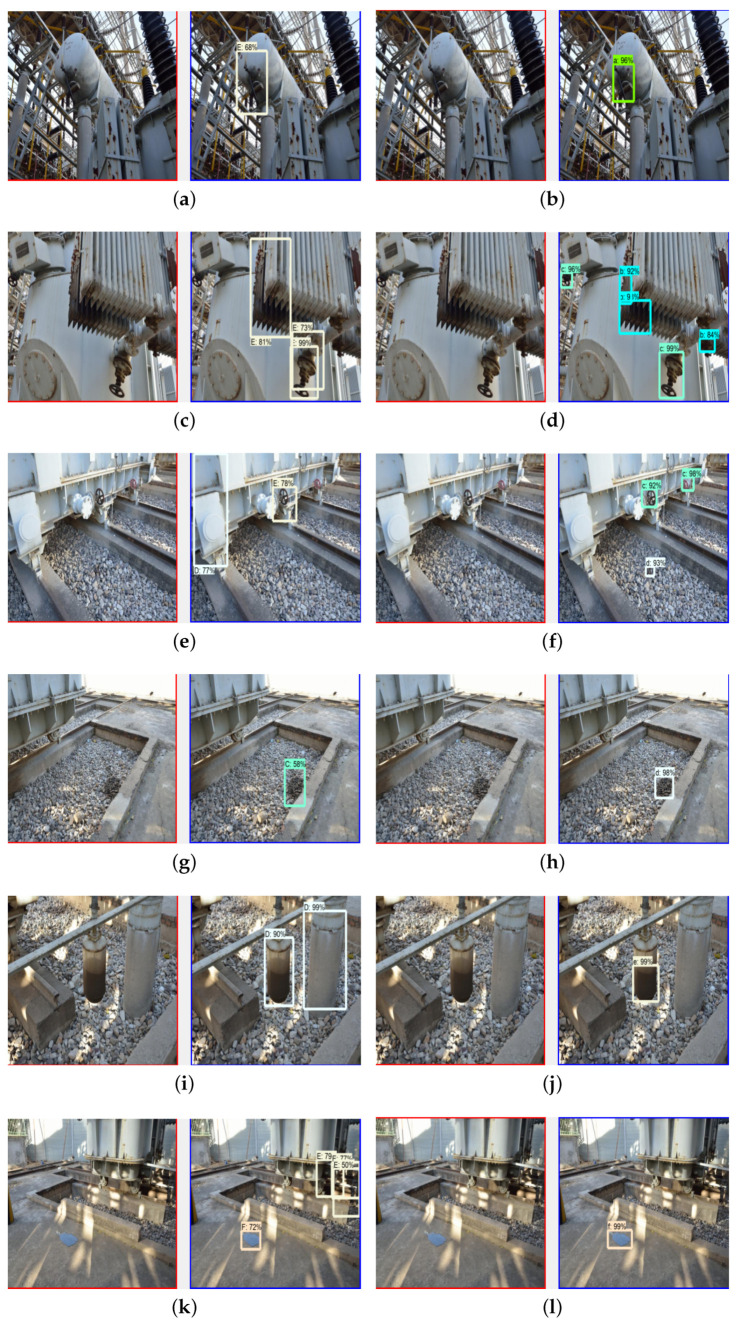
Comparison of test results before and after improvement in oil leakage detection for six types of equipment in real scenarios: (**a**) tank detection before model improved; (**b**) tank detection after model improved; (**c**) heat sink detection before model improved; (**d**) heat sink detection after model improved; (**e**) valve detection before model improved; (**f**) valve detection after model improved; (**g**) stone detection before model improved; (**h**) stone detection after model improved; (**i**) column detection before model improved; (**j**) column detection after model improved; (**k**) ground detection before model improved; (**l**) ground detection after model improved.

The performance of the improved model was further tested for oil leakage detection in six different types of substation equipment. Comparisons of the mAP of different models in different categories are shown in Table 5. The comparison of the test results before and after the improvement for six types of equipment in real scenarios are shown in Figure 10.

As shown in Figure 10 and Table 5, the oil leakage defect detection results of the six types of equipment showed that after the network structure improvement, the mAPs of all categories achieved better effectivenes. The proposed FRRNet101-c model in this paper achieved a detection mAP of 93% and 97% on the small objects of oil leakage from heat sinks and oil on stones, which is an improvement of 12% and 10%, respectively, compared with the unimproved FRRNet101-aug model. In addition, the FRRNet101-c model’s oil leakage detection effectiveness mAP achieved 99% in the remaining four categories.

To sum up, the FRRNet101-c model improved the mAP by 6.3% in real-time testing, especially in the detection of small objects, where the mAP improved by 12%, and the mAP of the remaining categories of detection accuracy reached 99%. Therefore, the FRRNet101-c model has the best effectiveness for substation equipment oil leakage detection and can provide accurate information about substation equipment oil leakage defects for substation inspection robots.

### 4.5. Implementation of Oil Leakage Decision-Making Scheme

In order to implement the substation equipment oil leakage detection method in an intelligent inspection robot, it is necessary to complete the training and testing of the model to transplant the hardware platform, which is the hardware platform construction stage. Considering the limitations of storage and computing power, a standalone embedded platform with a USB 2.0 camera was used and connected with an embedded industrial resistive touch screen for better performance. The embedded development board adopted the Raspberry Pi 3B+ development board (1 G). The Raspberry Pi 3B+ development platform features the Ubuntu MATE operating system and the TensorFlow deep learning framework.

**Figure 11:**
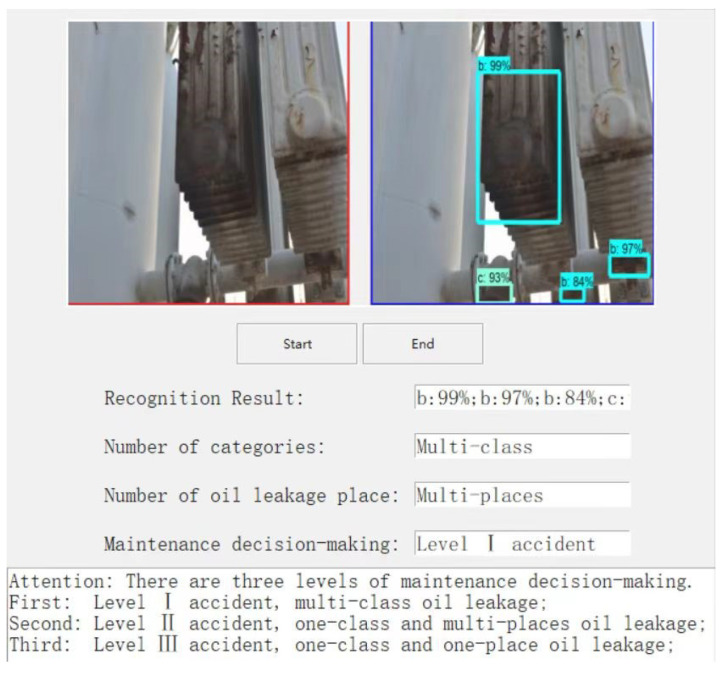
Level I accident.

**Figure 12:**
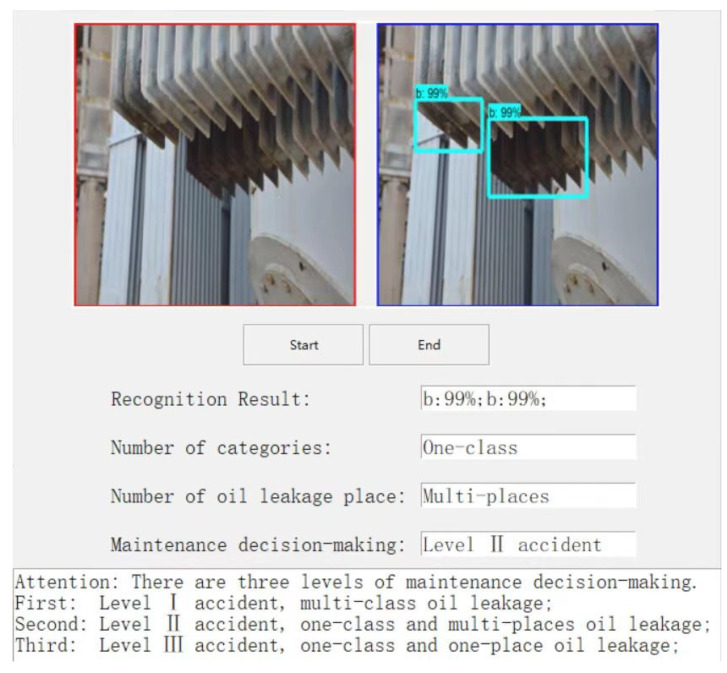
Level II accident.

**Figure 13:**
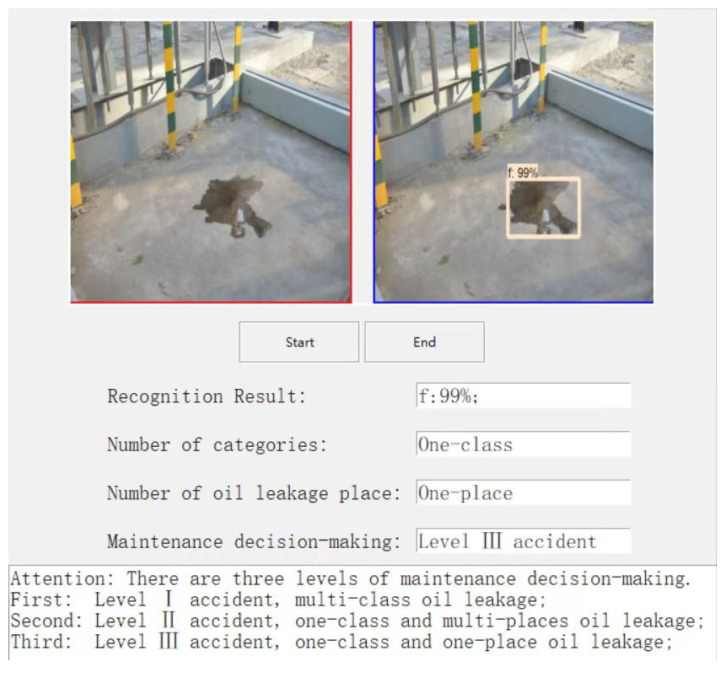
Level III accident.

To further validate the oil leakage detection method for substation equipment in an intelligent inspection robot, the completed trained and tested model was first ported to an embedded platform for detection of a substation equipment oil leakage scenario. Then, according to the number of oil leakage categories in the detection results and the number of oil leakage locations in a single category to determine the severity of oil leakage accidents, the detection results were automatically converted into decision-making recommendations within the algorithm. The decision recommendations were classified into three categories, including “Level I accident”, “Level II accident”, and “Level III accident”, as shown in Figure 11, Figure 12 and Figure 13. Finally, the decision recommendations and recognition results were uploaded to the host computer and displayed on the GUI interface, providing guidance information for substation staff to deal with equipment oil leakage strategies.

## 5. Conclusions and Future Work

This paper proposes a small object detection method for oil leakage defects for substation equipment, and designs an oil leakage decision-making scheme combined with an intelligent inspection robot. The substation oil leakage images used in this paper are collected via an inspection robot with a camera on board. Firstly, through analyzing the performance of several object detection networks in a public dataset, a model suitable for the oil leakage detection of substation equipment using inspection robots is selected and improved. Furthermore, based on the selected feature extraction network, the Faster-RCNN model of Resnet-101, adopting the improvement method of cancelling the downsampling and replacing the convolution kernel effectively reduces the loss of information in the original image, especially the loss of information about small objects. The improved model is referred to as the FRRNet101-c model. Finally, an oil leakage decision-making scheme is designed that can classify oil leakage accidents into three levels and show the accident levels through the GUI interface, providing guidance information for substation workers to deal with equipment oil leakage strategies. After experimental verification, the proposed method in this paper can effectively detect the oil leakage defects of substation equipment, which greatly improves the intelligence of oil leakage detection in substation equipment.

In future work, we will consider whether using generative adversarial networks to generate synthetic oil leakage images via data augmentation work can improve model detection accuracy. At the same time, the current hot transformer structure will be introduced, and whether it can improve the detection accuracy of small objects will be assessed.

## Figures and Tables

**Table 1:** Architecture of the FRRNet101-c network.

Serial Number	Name	Parameter	Output Size
1–3	Convolution layer	$\left[\begin{array}{c} 3\times 3,64\\ 3\times 3,64\\ 3\times 3,64 \end{array}\right],stride1$	112×112
4–12	Convolution layer	$\left[\begin{array}{c} 1\times 1,64\\ 3\times 3,64\\ 1\times 1,256 \end{array}\right]\times 3$	56×56
13–24	Convolution layer	$\left[\begin{array}{c} 1\times 1,128\\ 3\times 3,128\\ 1\times 1,512 \end{array}\right]\times 4$	28×28
25–93	Convolution layer	$\left[\begin{array}{c} 1\times 1,256\\ 3\times 3,256\\ 1\times 1,1024 \end{array}\right]\times 4$	14×14
94–102	Convolution layer	$\left[\begin{array}{c} 1\times 1,512\\ 3\times 3,512\\ 1\times 1,2048 \end{array}\right]\times 4$	7×7
103	Classifier layer	average pool, 1000-d fc, Softmax	1×1

**Table 2:** Comparison of the substation equipment oil leakage dataset before and after data augmentation.

	Total	Training Set	Test Set	Proportion of Labels
Original data	438	399	39	1:0.4:1.3:0.6:0.8:0.9
First data augmentation	560	507	53	1:0.9:1.1:0.9:1:0.9
**Second data augmentation**	**3300**	**3000**	**300**	**1:0.9:1.1:0.9:1:0.9**

**Table 3:** Comparison of model size, speed, and mAP.

Model	Model Size (MB)	Speed (ms)	COCO mAP
**Faster-RCNN-Resnet-101**	**187**	**106**	**32%**
Faster-RCNN-Inception-Resnet-V2	235.2	620	37%

**Table 4:** Comparison of mAP for training and testing of different models and training time consumed.

Model	Training mAP	Testing mAP	Training Time
FRRNet101	71.98%	68.93%	20 h 18 m 58 s
FRRNet101-aug	91.54%	89.13%	20 h 34 m 28 s
FRRNet101-a	93.07%	91.26%	21 h 02 m 09 s
FRRNet101-b	96.76%	94.52%	21 h 37 m 52 s
**FRRNet101-c (Ours)**	**97.76%**	**95.43%**	**23 h 52 m 18 s**

**Table 5:** Comparison of different models for different categories of mAP.

Model	Tank	Heat Sink	Valve	Stone	Column	Ground
FRRNet101	85%	51%	75%	62%	78%	81%
FRRNet101-aug	98%	81%	94%	87%	93%	96%
FRRNet101-a	98%	85%	95%	90%	94%	96%
FRRNet101-b	99%	92%	98%	94%	98%	99%
**FRRNet101-c (ours)**	**99%**	**93%**	**99%**	**97%**	**99%**	**99%**

## Data Availability

The data used to support the findings of this study are available from the corresponding author upon request.
